# Biomechanical Characterization of Metacarpal Fixation: Internal Load Determination and Evaluation of a Novel Adhesive Osteosynthesis

**DOI:** 10.1002/jor.70027

**Published:** 2025-07-27

**Authors:** Peter Schwarzenberg, Gordian Banzer, Guillaume Patt‐Lafitte, Jérôme Schlatter, Daniel J. Hutchinson, Michael Malkoch, Peter Varga, Tatjana Pastor

**Affiliations:** ^1^ AO Research Institute Davos Davos Graubünden Switzerland; ^2^ Mines Saint‐Etienne, Univ Jean Monnet, INSERM, UMR 1059 Sainbiose Saint‐Etienne France; ^3^ Department of Fibre and Polymer Technology KTH Royal Institute of Technology Stockholm Sweden; ^4^ Department for Plastic and Hand Surgery Inselspital University Hospital Bern, University of Bern Bern Switzerland; ^5^ Departmentof Traumatology and Orthopaedics Bürgerspital Solothurn Solothurn Switzerland

**Keywords:** biomechanics, finite element modeling, metacarpal fracture, osteosynthesis

## Abstract

Metal plates are commonly used for the fixation of metacarpal fractures, but are associated with complications such as tendon adhesions that impair finger mobility. AdhFix, a novel osteosynthesis material, may reduce these complications, but its biomechanical performance in the metacarpal region remains untested. This study aimed to evaluate internal bending moments during postoperative rehabilitation and assess the biomechanical potential of AdhFix in treating comminuted metacarpal shaft fractures using a human cadaveric model. In five cadaveric hands, unstable shaft fractures of the second, third, and fourth metacarpals were simulated using a 3 mm osteotomy gap and stabilized with polyether ether ketone (PEEK) plates and stainless‐steel cortical screws. A custom 3D‐printed guide ensured accurate osteotomy and screw positioning. Tendon loading was simulated by pulling flexor digitorum profundus tendons until fingertip‐to‐palm contact was achieved, followed by wrist flexion using 100 N of force on the flexor carpi radialis and ulnaris tendons. Bending of the fixation was captured using stereographic imaging, and internal bending moments were calculated via finite element modeling. After removal of the PEEK plate, the procedure was repeated using AdhFix for fixation. Maximum internal bending moments were 6.14 ± 2.03 Nmm during finger flexion and 3.37 ± 1.64 Nmm during wrist flexion. AdhFix demonstrated mechanical integrity under both conditions with no observed failure.

**Statement of Clinical Significance:**

AdhFix may provide a mechanically stable alternative to conventional plates during early rehabilitation, potentially reducing tendon adhesions and preserving finger mobility.

## 1 Introduction

Metacarpal fractures account for up to 33% of all hand fractures [1, 2, 3]. Conservative treatment is usually sufficient for fractures that are not comminuted or displaced. However, in the case of comminution or dislocation, surgical intervention is generally recommended. Surgical treatment methods vary, with k‐wiring, intramedullary screw fixation, and plating accounting for only some of the many options [4, 5, 6, 7]. In transverse and short oblique metacarpal shaft fractures, plate fixation is the preferred treatment option [5]. Nevertheless, a possible downside of plate fixation is soft tissue adhesion as well as disturbing hardware, leading to irritation of the extensor tendons. Additionally, metal plates become compromised in specific fracture patterns due to their constrained adaptability. Due to these reasons, new osteosynthesis materials and methods are being developed [8, 9]. AdhFix, a novel osteosynthesis technique, has been developed to address these clinical limitations. The AdhFix solution utilizes a light‐curable resin composite for this new osteosynthesis method [10], composed of high concentrations of hydroxyapatite combined with triallyl and trithiol triazine‐trione (TATO) monomers, and anchored to the bone surface with traditional metal screws [11]. When exposed to high‐energy visual visible (HEV) light, the initiator is decomposed, forming initiator that enables the monomers in the composite undergo rapid and efficient thiol‐ene coupling chemistry thereof producing a hard crosslinked network [11, 12]. This results in a plate‐like construct that can be shaped precisely in situ before curing, offering the surgeon the ability to shape an individual construct for each fracture. Furthermore, fracture fixation only needs to be stable enough to allow early motion exercises, avoiding finger stiffness [13, 14]. AdhFix has already been shown in prior biomechanical studies that it offers sufficient stability compared to conventional metallic plates [15, 16]. It is hypothesized that AdhFix demonstrates a comparable biomechanical behavior as metallic plates in metacarpal fractures under low loading conditions corresponding to early rehabilitation exercises and may be considered as a valid alternative. Therefore, the aim of this study was to investigate the biomechanical load within the metacarpal during non‐load bearing rehabilitation exercises and test the performance of AdhFix in comminuted metacarpal shaft fractures in a human cadaveric bone model.

## 2 Methods

### 2.1 Overview

This study determined the internal loads applied to plate osteosyntheses in metacarpal fractures under simulated rehabilitation exercises based on methods established in a previous study [16]. The evaluation technique relied on a rectangular plastic (polyether ether ketone, PEEK) plate with known biomechanical properties to fix a 3 mm gap osteotomy at midshaft. Optical markers were fixed to the bone on each side of the fracture, proximally and distally to the PEEK plate. Using a stereographic motion tracking camera system, the displacements of the bone fragments were calculated while undergoing the simulated fingertip‐to‐palm or wrist flexion rehabilitation exercises. These displacements were then used as the inputs into construct‐specific finite element (FE) models, where the internal loads in the plastic plate could be calculated. Complete details on the established method can be found in a previous publication [16].

After the determination of the internal loads in the osteosynthesis, AdhFix was applied to the osteotomized bones and tested under the same conditions as a validation that the novel AdhFix fixation would survive the simulated rehabilitation exercises.

### 2.2 Specimen Information

Five human cadaveric arm donors (above elbow) with an average age of 42.6 years (range 34–50 years) were utilized in this study (Table 1). All donors gave their informed consent inherent within the donation of the anatomical gift statement during their lifetime (Science Care). There was no evidence of prior bone fractures or other injuries to the hands in the specimens. A high‐resolution peripheral quantitative computer tomography scan (HR‐pQCT, XtremeCT II; Scanco Medical AG) was conducted on each specimen before starting any experimental procedures. Each scan had an X‐ray voltage of 60 kVp, X‐ray current of 0.90 mA, and an isotropic voxel size of 82 µm. This scan provided a quantitative measurement of bone geometry and bone mineral density of the intact metacarpals used in the FE model.

**Table 1:** Detailed information for each hand specimen.

Hand ID	Side	Sex	Age	Height		Weight		BMI
[‐‐]	[Left/Right]	[Male/Female]	[years]	[in]	[cm]	[lbs]	[kg]	[‐]
Hand 1	Left	Male	41	67	170.2	181	82.1	28.4
Hand 2	Right	Male	41	67	170.2	181	82.1	28.4
Hand 3	Right	Male	50	71	180.3	210	95.3	29.3
Hand 4	Right	Male	47	67	170.2	174	78.9	27.3
Hand 5	Right	Male	34	72	182.9	192	87.1	26.5
		**Average**	42.6	68.8	174.76	187.6	85.1	27.98
		**Std**	5.54	2.23	5.65	12.60	5.74	0.97

### 2.3 Surgical Procedure

**Figure 1:**
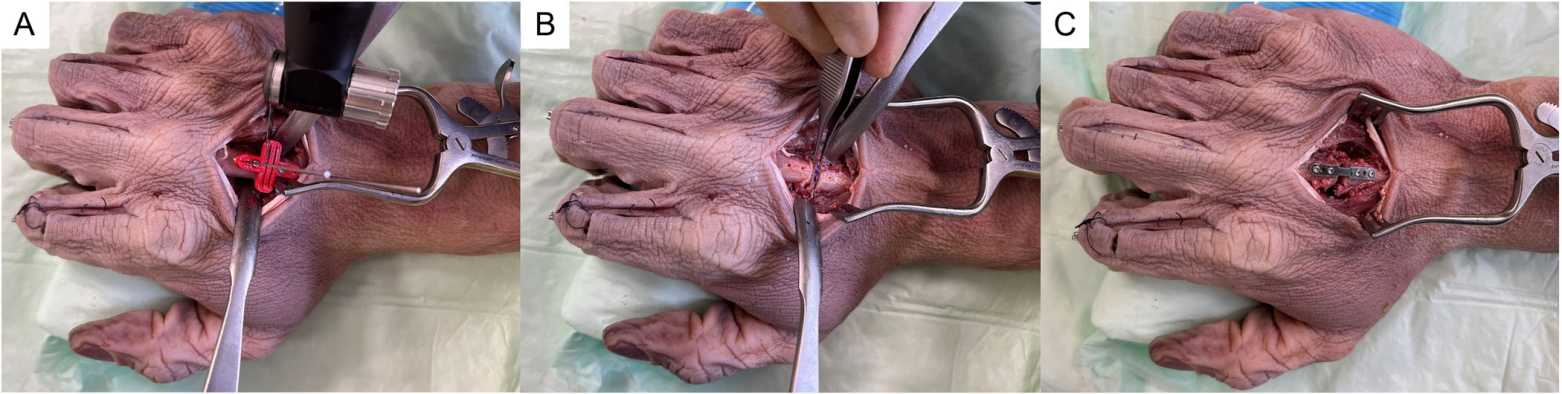
Surgical procedure showing (A) surgical access and the osteotomy cutting guide placement, (B) the completed osteotomy and pilot holes for cortical screws, and (C) the PEEK measurement plate fixed with cortical screws.

The cadaver specimens were previously used in a study that employed an osteotomy in the diaphysis of the proximal phalanx [16]. These osteotomies were stabilized with a custom 3D printed phalangeal‐specific spacer and two 1.8 mm diameter Kirschner wires (K‐wires, double tip, 1.8 mm × 150 mm, Johnson & Johnson MedTech) to accomplish sufficient fixation stability in each proximal phalanx. Furthermore, the deep flexor tendons (flexor digitorum profundus (FDP)) were evaluated by the attending surgeon to ensure proper finger flexion, since they also were used in the previous investigation. In this previous investigation, the tendons for the 4th digit in Hand 2 were severed, rendering this digit unusable for this study. The flexor carpi ulnaris (FCU) and the flexor carpi radialis (FCR) tendons were then located and isolated since they are the strongest wrist flexor tendons [17]. To ensure a balanced wrist flexion, the wrist was fully flexed and required to return to a neutral position with no radial or ulnar deviation. A 1.5 mm braided loop steel cable was then attached to each flexor tendon with suture material (Covidien Polysorb Braided Absorbable Suture, size 3‐0). To gain access to perform the osteotomy on the metacarpal bones, a dorsal longitudinal incision was made. The extensor tendons were either held to the side (for the 2nd metacarpal) or a tendon split was performed (for the 3rd and 4th metacarpal; Figure 1A).

For a precise osteotomy gap of 3 mm at the mid diaphysis, 3D printed cutting guides were designed and produced for each metacarpal for each finger and specimen individually (Figure 1A). The 3D printed cutting guides contained four screw holes spaced at 5 mm apart, two on each side of the osteotomy gap and at 5 mm from the center of the osteotomy. Additionally, the cutting guides contained two slits for the cutting blade (Figure 1A). The cutting guides were secured to the bone with 0.8 mm K‐wires in the outer holes on either side of the fracture. Next, 1.1 mm bicortical pilot holes were drilled in the inner holes, and 1.5 mm cortical screws (cortex screw stardrive, Johnson & Johnson MedTech) were inserted into the pilot holes. A 3 mm osteotomy gap was cut using the cutting guide's slits with a 0.6 mm‐thick saw blade (Johnson & Johnson MedTech) using a Unium^TM^ machine (Johnson & Johnson MedTech). Afterwards, the K‐wires were removed, and additional 1.1 mm pilot holes were drilled in the outer holes in the cutting guide (Figure 1B). The remaining inner screws and cutting guide were then removed. The osteotomies were secured with a polyether ether ketone (PEEK) plate (24.25 × 4.25 mm) and four bicortical 1.5 mm screws (Figure 1C).

Following the surgical procedures, a 3D image of each fixation construct was taken with a 3D C‐Arm (Ziehm Vision RFD 3D, Ziehm Imaging GmbH) to measure the postoperative position of the bone fragments and hardware. The 3D image was taken with a field of view diameter of 200 mm and a resulting voxel size of 0.39 mm. To better visualize the PEEK plate, a thin layer of zinc paint was applied to the plate before scanning.

### 2.4 Biomechanical Evaluation: Internal Loads

**Figure 2:**
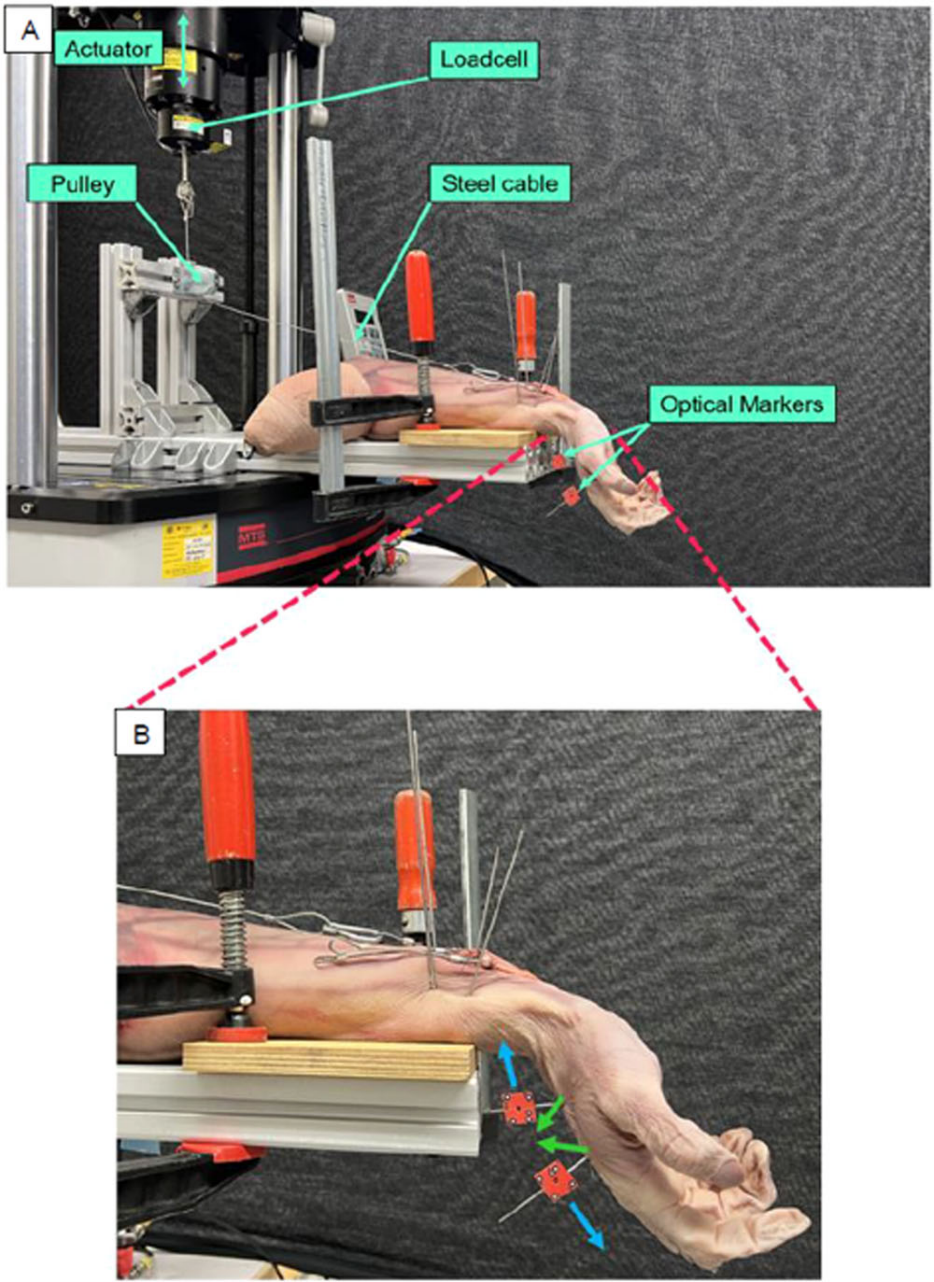
(A) Setup for biomechanical experiment. (B) Zoomed in for better recognition of the relevant test object. Blue arrows indicate displacement measured by optical motion tracking. Green arrows demonstrate the calculated reaction forces of the Finite Element model.

Biomechanical testing was performed on an electrodynamic testing machine equipped with a 3 kN load cell (Acumen; MTS, Eden Prairie). The test setup and loading protocol were adopted from a previous study (Figure 2) [16].

Two 1.6 mm K‐wires were first inserted into the metacarpal bone, one proximal and one distal to the osteotomy gap. Optical markers were then attached to the K‐wires for optical motion tracking (Figure 2B blue arrows). Each specimen was secured by two K‐wires through the radius and ulna, 4 cm proximal to the wrist crease, to a wooden board, which was fixed to the base on the testing machine. The hand was placed in an upward position. The braided steel cables were attached to the testing machine via a pulley system in such a manner that the vertical load of the machine could be translated into a force vector along the axis of the forearm (Figure 2). Two tests were performed for non‐weight bearing rehabilitation exercises, fingertip to palm (Test 1) and flexion of the wrist (Test 2).

Test 1 was performed with the wrist in a neutral position and subsequently flexing each digit consecutively by loading the respective FDP tendon until the fingertip reached the palm of the hand, using the testing machine's actuator at a strain rate of 1 mm/s. For Test 2, the wrist was flexed by loading the FCU and FCR tendons simultaneously until a force of 100 N was reached on the testing machine. The testing machine recorded the axial translation, and the associated force applied during these movements in both tests. A stereographic camera system, the Aramis SRX (GOM GmbH), was used to track the movement of the bone fragments and, consequently, the deformation of the PEEK plate during the flexion process. Each digit was tested five times, with trials 3–5 used in the data analysis to account for any setup artifacts. After biomechanically testing each metacarpal, the PEEK plate was removed, and the osteotomy was securely stabilized using a custom‐designed and manufactured metal plate. To provide a rigid stability, a 3D printed spacer was placed into the 3 mm osteotomy gap. Further details of this established testing protocol can be found in a previous publication [16].

**Figure 3:**
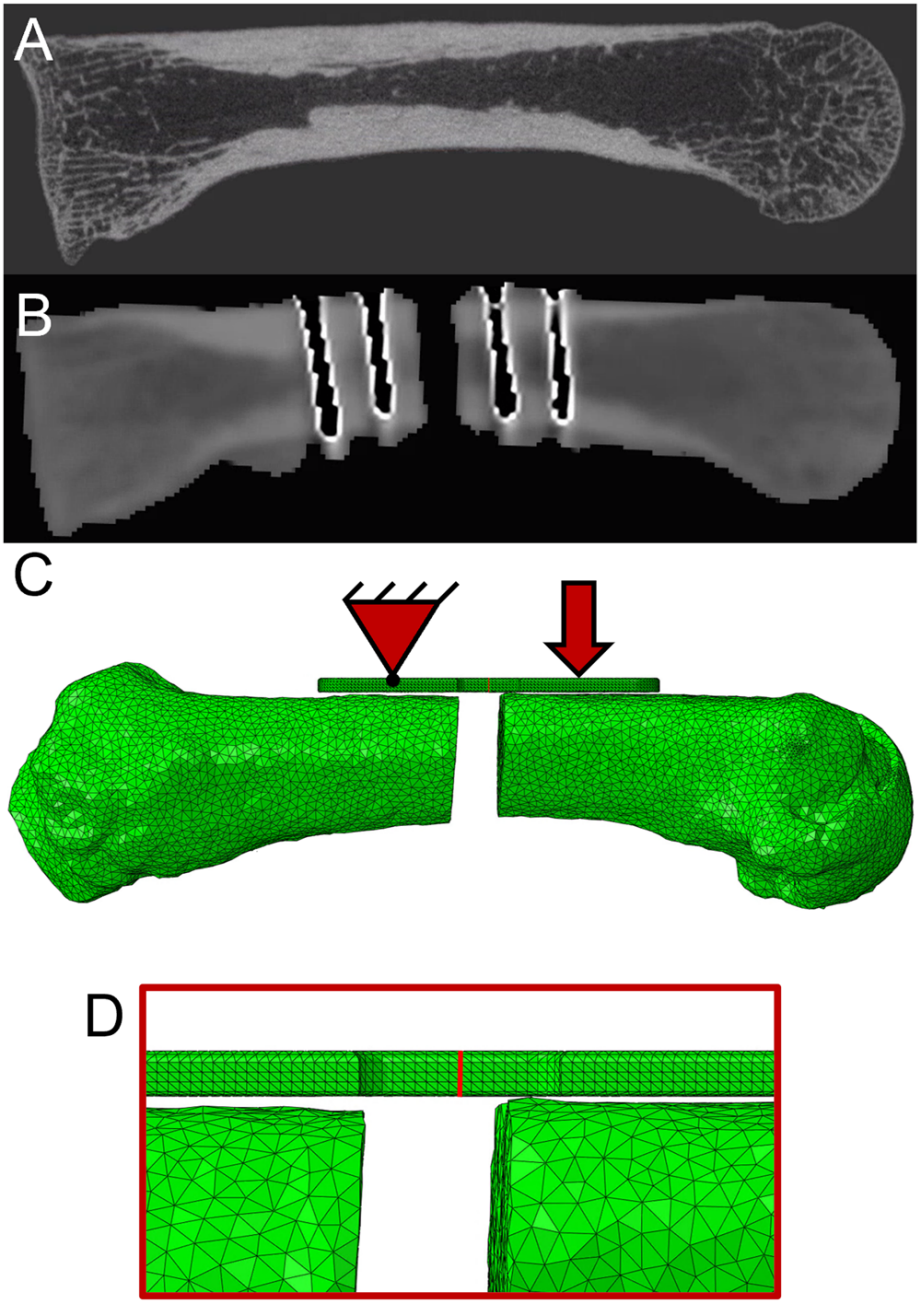
(A) Preoperative HRpQCT scan of representative sample. (B) Postoperative 3D C‐Arm image of metacarpal with osteotomy and screws. (C) Finite element model with boundary conditions of a pin support and applied displacement. (D) Zoomed‐in view of region of interest, highlighting cut plane of the plate where the bending moment is calculated.

To create specimen‐specific FE models of the metacarpal bones, the preoperative HRpQCT scans (Figure 3A) were first aligned with the postoperative 3D C‐Arm images (Figure 3B) in Amira 3D (v2021, Thermo Fisher Scientific). Both sets of images were segmented, and a spatial registration was conducted. The PEEK plate was aligned with the segmented postoperative metacarpal bone using the screw heads as reference markers. The preoperative HR‐pQCT image of the bone was then registered to the postoperative 3D C‐arm image of the construct. The osteotomy and hardware positions were taken from the postoperative image. This methodology ensured accurate alignment of metacarpal bones while retaining the heterogeneous underlying structures for biomechanical analysis.

Finite element model preparation was performed in Simpleware (v17, Simpleware Ltd.), integrating segmented greyscale images and PEEK plate geometry. The meshing utilized quadratic tetrahedral elements with optimized parameters from a previous convergence study, which determined convergence as less than a 5% difference in bending moment between mesh sizes [16], achieving minimal error with computational efficiency. This included a minimum edge length of 0.25 mm, a maximum error of 0.082 mm, and a maximum edge length of 1 mm with a 0.25 mm maximum edge length in a refinement area in and around the plate. Screw positions and corresponding node sets were established for accurate representation of bone‐plate and screw interactions. These configurations provided a robust foundation for subsequent FE simulations (Figure 3C).

The FE analysis of a PEEK plate fixation system was conducted using Abaqus CAE (v2021, Dassault Systems), incorporating meshed proximal and distal metacarpal bone fragments and a CAD model of the plate. Reaction forces and bending moments were analyzed along all axes of a defined coordinate system aligned with the anatomical orientation of the hand. Special focus was given to the bending moment around the *z*‐axis, defined as perpendicular to the plane of normal hand flexion (i.e., orthogonal to the sagittal plane). This out‐of‐plane moment reflects torsional and bending loads experienced by the fixation system in the horizontal plane during physiological movement, providing insight into its mechanical stability in metacarpal fracture fixation. A normalized distance was taken across each testing data set, from 0 to 1, spanning the range of motion. In the fingertip to palm test (Test 1) the full range (0–1) was analyzed. In the wrist flexion test (Test 2), only the first half (0–0.5) was analyzed as the second half appeared to overextend the natural flexion and resulted in unnatural results.

### 2.5 Biomechanical Evaluation: AdhFix

**Figure 4:**
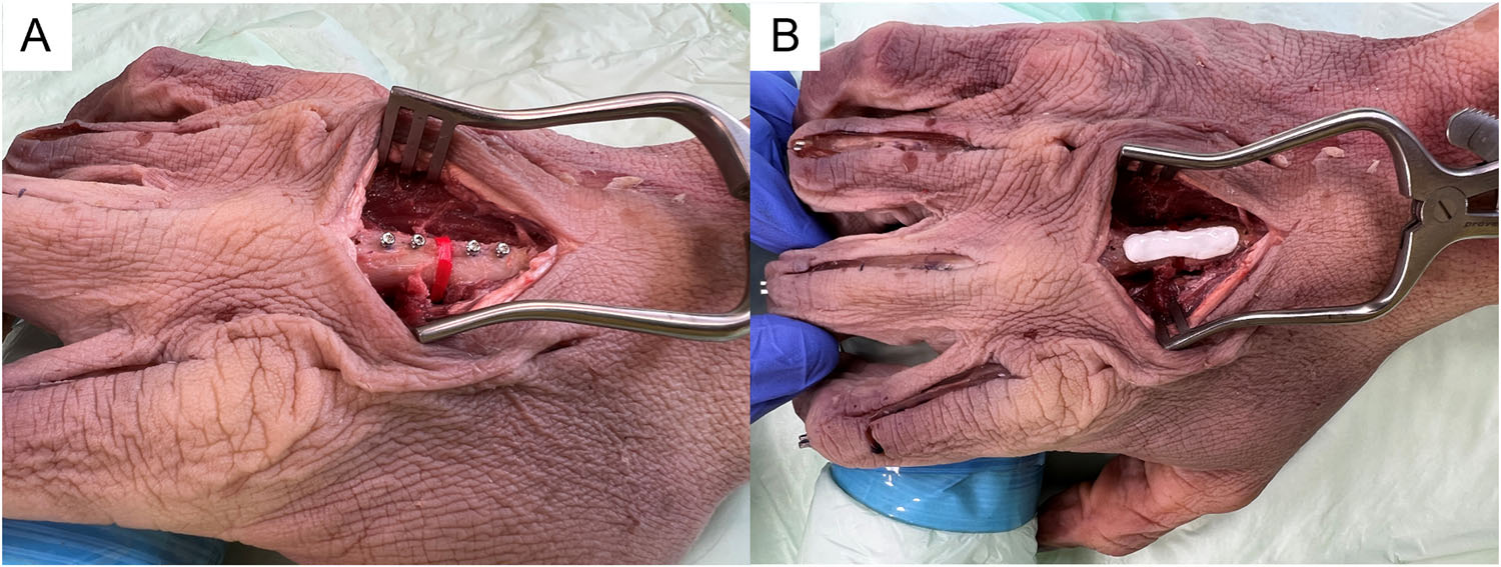
AdhFix application Procedure. (A) Insertion of the specimen‐specific 3D printer spacer to maintain a 3 mm osteotomy gap. (B) Completed AdhFix patch on a metacarpal.

Following the mechanical testing to measure the internal loads on the metacarpal bones, AdhFix was applied to the same metacarpal osteotomies (Figure 4). The purpose of the following tests was to evaluate the AdhFix patch's durability during the fingertip to palm and wrist flexion rehabilitation exercises. The primary goal was to determine if the AdhFix patch would fail during the rehabilitation exercises, as indicated by fracture or breakage (pass/fail regime). For this purpose, the metacarpal bones, which were not tested, were stabilized as in the previous section. AdhFix was applied according to the described protocol by Colding‐Rasmussen [18]. Previous studies have shown that once the thiol‐ene composite is cured, its reported flexural strength is 69 ± 3 MPa and its flexural modulus is 6.6 ± 0.2 GPa [11]. Four screws were inserted into the pre‐drilled holes until the threads were fully engaged in the bone, but the screw heads were left unseated and not tightened, ensuring that no compressive force was applied to the surrounding material at this stage. The uncured resin composite was applied around each screw, which was then tightened. A HEV light (Bluephase PowerCure LED lamp, Ivoclar Vivadent Clinical) was then used to cure the composite with two pulses of 5 s duration per 50 mm^2^ area at a light intensity of 2000 mW/cm^2^. Afterwards, two additional composite layers were sequentially added and cured. Finally, Test 1 and Test 2 were repeated as described for the internal loads study.

### 2.6 Statistical Analysis

Descriptive statistics, pair‐sample T‐tests, and one‐way ANOVAs were conducted in SPSS (IBM SPSS Statistics, V27, IBM). In the pair‐sample T‐Test, outliers were retained as they were assumed to be meaningful results. If Levene's test revealed non‐homogeneity of variances, then a Welch ANOVA with a Games Howell post hoc correction was used. Normality was determined by a Shapiro–Wilk test. The level of significance was set to 0.05 for all tests.

## 3 Results

### 3.1 Internal Loads

Similar trends in the average bending moments were observed among the five hands throughout Test 1 (fingertip to palm) as well as Test 2 (wrist flexion), which are summarized in Table 2. There were noticeable mechanical variations between the digits in both simulated rehabilitation tests. The average ± standard deviation for the maximum bending moments in Test 1 were 6.14 ± 2.03 Nmm, and in Test 2 were 3.37 ± 1.64 Nmm and were significantly different as shown by a paired‐sample *T*‐Test (*p* < 0.001).

**Table 2:** Average bending moment and standard deviation over the normalized movement of biomechanical tests for both tests and each metacarpal bone.

Average bending moment						
Step [‐]	Test 1: Fingertip to palm			Test 2: Wrist flexion		
	2nd [Nmm]	3rd [Nmm]	4th [Nmm]	2nd [Nmm]	3rd [Nmm]	4th [Nmm]
0	0 ± 0	0 ± 0	0 ± 0	0 ± 0	0 ± 0	0 ± 0
0.2	0.25 ± 0.23	0.09 ± 0.13	0.19 ± 0.22	0.65 ± 0.46	0.42 ± 0.25	0.24 ± 0.18
0.4	0.49 ± 0.80	0.29 ± 0.26	0.92 ± 0.72	2.26 ± 1.30	1.67 ± 0.64	0.83 ± 0.48
0.6	1.39 ± 1.08	0.76 ± 0.59	1.69 ± 0.68	3.56 ± 1.67	2.59 ± 0.82	1.38 ± 0.61
0.8	4.09 ± 0.75	1.56 ± 1.30	2.53 ± 1.22	4.25 ± 1.45	3.03 ± 0.96	1.86 ± 0.69
1	8.57 ± 1.06	4.71 ± 1.02	4.88 ± 0.51	4.77 ± 1.34	2.98 ± 1.42	2.13 ± 0.68

**Figure 5:**
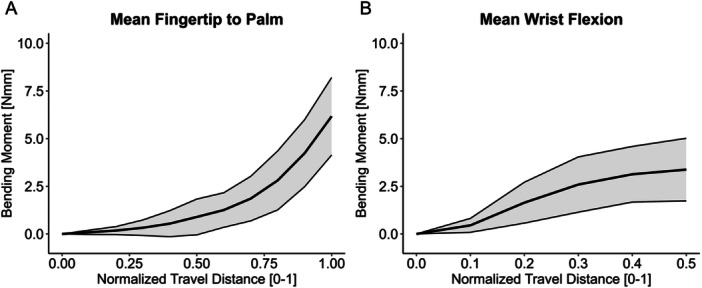
Average bending moment curves for all specimens with standard deviation region shaded. (A) Fingertip to palm test (Test 1). (B) Wrist flexion test (Test 2).

In Test 1, a nonlinear increase in the bending moment was seen as the normalized travel distance increased, resulting in an exponential‐like curve (Figure 5A). In contrast, Test 2 resulted in the bending moment increasing at the beginning of the test and stabilizing as the travel distance increased (Figure 5B).

With the exception of two groups (Test 1, Hands 1 and 3: *p* = 0.001 and 0.027; Test 2, Hands 1 and 3: *p* = 0.001 and 0.029), all groups exhibited normal distribution assessed by the Shapiro–Wilk test. Considering this, a one‐way ANOVA was still used due to its resiliency to deviations from normalcy [19]. The metacarpal bones were divided into three groups for additional analysis: the second metacarpal (*n* = 15), the third metacarpal (*n* = 15), and the fourth metacarpal (*n* = 12). Levene's test revealed non‐homogeneity of variances in both tests (Test 1 and Test 2); therefore, a Welch ANOVA with a Games–Howell post hoc correction was used.

**Figure 6:**
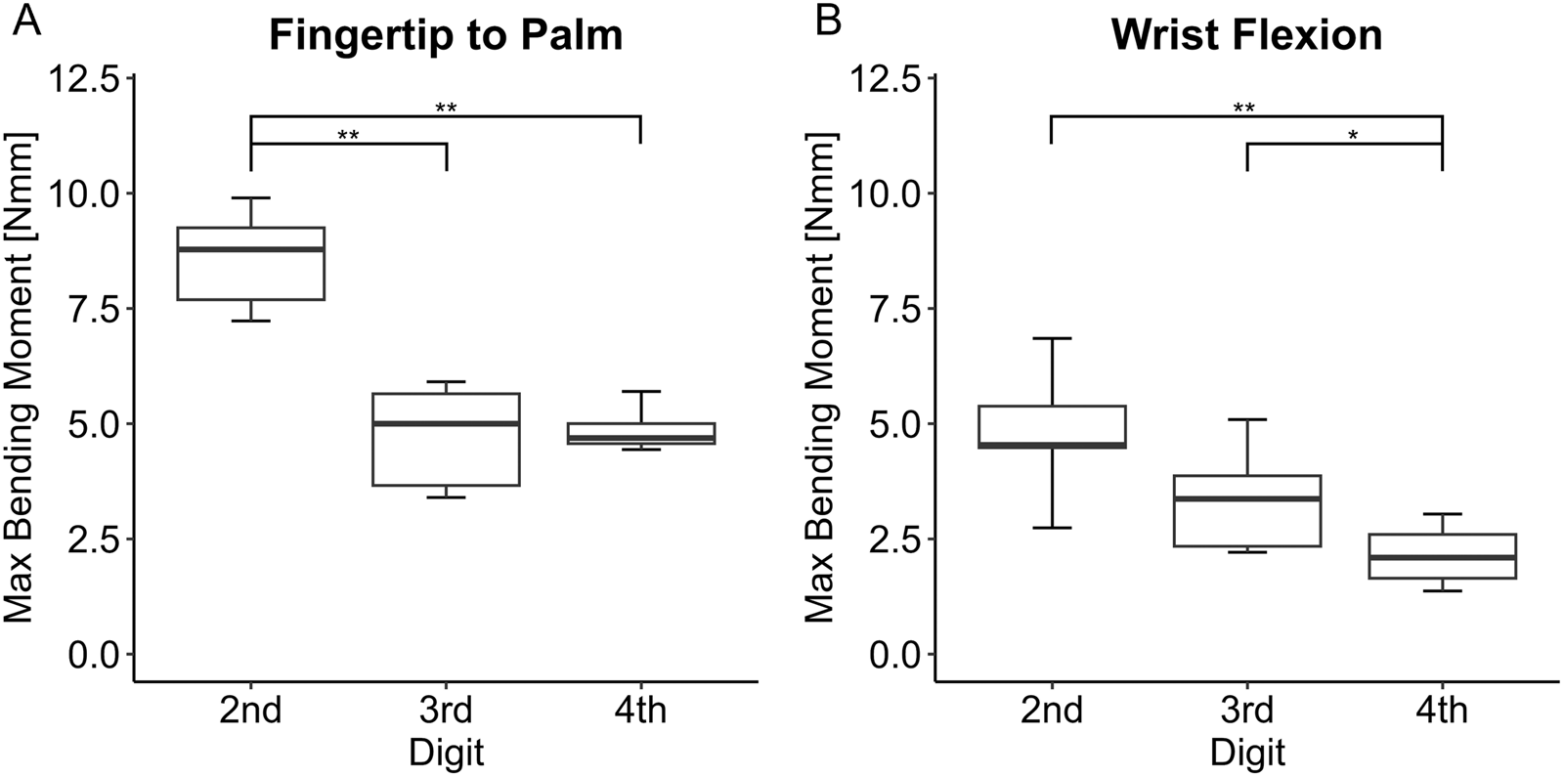
(A) Box plots of maximum bending moment in fingertip to palm test (Test 1) grouped by digit. (B) Box plots of maximum bending moment in wrist flexion test (Test 2) grouped by digit. *p < 0.05, **p < 0.001.

The one‐way ANOVA showed significant differences between the digits in both tests (Figure 6). In Test 1, the 2nd digit was significantly different from the 3rd and 4th digits (Figure 6A). Conversely, in Test 2, the 4th digit was significantly different from the 2nd and 3rd digits (Figure 6B).

### 3.2 AdhFix

All AdhFix samples successfully withstood the simulated rehabilitation exercises in Test 1 and Test 2. There were no signs of fracture across the fracture gap or around the screws. Additionally, there were no adhesion issues within the composite, or its fit to the bone.

## 4 Discussion

The current study determined internal bending moments applied to osteosynthesis devices during the stabilization of comminuted metacarpal fractures, particularly during rehabilitation activities without external loading. When evaluating the effectiveness of new or pre‐existing osteosynthesis devices developed to treat metacarpal fractures, these calculated internal loads are crucial. Furthermore, this investigation was able to show that AdhFix was capable of tolerating metacarpal fracture postoperative rehabilitation protocols, which is encouraging for its future development. Findings of this investigation reveal an exponential increase in bending moments during fingertip‐to‐palm motion, influenced by the complex anatomical interactions of phalanges and metacarpals. Distinct load distributions were observed among metacarpals, with the second metacarpal experiencing higher bending moments (8.57 ± 1.06 Nmm) due to its position in the hand. The third and fourth metacarpal bone are stabilized by the surrounding metacarpals explaining their close values at 4.71 ± 1.02 and 4.88 ± 0.51 Nmm, respectively, whereas the second and fifth lie at the margin of the hand. Another reason is the more stable CMC‐III‐ and IV‐joints in contrast to the CMC‐II‐joint. Furthermore, due to the rotation of conics, the 4th and 5th metacarpals have not only a longitudinal axis movement but also a radial rotation movement towards the center of the hand [17, 20]. The wrist flexion test exhibited greater variability in bending moments, highlighting the intricate load dynamics of wrist structures. The mediocarpal and radiocarpal joints act as a cardan joint [21]. This type of joint has two axes, which are defined by the central cross element. This enables the transmission of a primary axis rotation to a second axis regardless of the angle between the two axes [22]. The developed methodology in the current investigation demonstrated high reproducibility and reliability in measuring bending moments, as validated by consistent trends and low standard deviations across trials.

The current investigation builds upon our previous study on internal loads in proximal phalanx fracture fixations during simulated rehabilitation exercises [16]. While the same methodology was applied—using stereographic tracking and finite element modeling—important differences emerged due to anatomical and functional variations between the phalanx and metacarpal bones. In the phalanx model, bending moments during fingertip‐to‐palm motion were similar, with a peak value of 6.78 ± 1.62 Nmm compared to 6.14 ± 2.03 Nmm in the metacarpal. However, in the 2nd digit, the metacarpal experiences larger moments of 8.57 ± 1.06 Nmm compared to 6.08 ± 1.08 Nmm in the phalanx. This difference may be attributed to the larger lever arm and during isolated finger flexion. In contrast, metacarpals benefit from additional structural support within hand and neighboring bones for the 3rd and 4th digits, with bending moments of 4.71 ± 1.02 and 4.88 ± 0.51 Nmm compared to 7.47 ± 1.52 and 6.79 ± 1.62 Nmm in the phalanx.

The study also indicates the robustness of AdhFix in withstanding non‐load‐bearing rehabilitation exercises, with all specimens enduring non‐weight‐bearing exercises. From a surgical perspective, metal plates are suitable for open reduction internal fixation in comminuted metacarpal shaft fractures as they demonstrate safe feasibility and stability [23]. However, they often lead to adhesion of the soft tissue and can be disturbing, leading to high implant removal rates [24]. There are many ongoing attempts to improve and develop new hardware for fracture fixation [25, 26, 27]. AdhFix has been developed to address these clinical problems [28]. The new osteosynthesis device has been evaluated in a prior investigation using a 3 mm fracture gap model in an ex vivo ovine proximal phalanx midshaft in 4‐point bending under quasi‐static loading [15]. In this case, the lowest bending moment to failure was 728 Nmm, with a mean maximum bending moment and associated standard deviation of 1220 ± 300 Nmm. Considering that the load applied to the osteosynthesis in a human metacarpal shaft fracture during conventional rehabilitation exercises were 6.14 ± 2.03 Nmm for fingertip to palm exercise and 3.37 ± 1.64 Nmm for wrist flexion exercise, AdhFix provided more than 100 times higher bending stability when tested monotonically to failure ex vivo in a ovine phalanx model [15]. Even though AdhFix's maximum bending moment has been shown to be inferior to that of metal plates [15], these findings imply that it may offer enough biomechanical strength to stabilize a metacarpal shaft fracture during fingertip‐to‐palm and wrist flexion rehabilitation exercises. Consequently, our study suggests that AdhFix may offer enough biomechanical stability in metacarpal shaft fractures to enable early rehabilitation and basic daily living activities. Further research is necessary to ascertain how well such a device performs under repetitive cyclic loading, mimicking the postoperative rehabilitation phase. Additionally, further investigations are necessary before AdhFix could be applied clinically.

Several limitations of the current study must be considered. First of all, in vivo circumstances following a fracture in a real human cannot be accurately replicated by a cadaveric bone model. Furthermore, working with human cadaver specimens involves ethical considerations, limited availability, and high costs, which constrained the sample size in this study. Additionally, all specimens were male, representing a further potential bias in the results. These limitations could influence the power of the statistical tests performed. Specifically, in this study, gap osteotomies were required for the method to calculate the internal forces and moments. Moreover, it is yet unclear which loading thresholds fixation structures must withstand in vivo, which remains a major problem with biomechanical investigations on the upper extremities [29], with this study only focused on one component of bending moment. A formal FE validation against experimental strain or deformation data has not been performed for the model being used. However, the modeling approach follows a previously established and peer‐reviewed method [16]. Additionally, the number of cycles that the constructs must withstand to achieve bone repair remains unclear. This study focused on the initial postoperative phase, representing a worst‐case scenario without the support of bone healing. However, future studies are needed to evaluate the fatigue properties of such fixation patches under clinically relevant cyclic conditions. Moreover, the AdhFix construct was custom‐made, leading to some variation in application, which might impact biomechanical behavior. Since the data did not show significant differences within the tested metacarpals, this aspect can be neglected.

Strengths of the current study lie especially in the use of a precise motion tracking system coupled with FE analysis. Thus, the findings provide valuable insights for optimizing osteosynthesis designs and tailoring patient‐specific rehabilitation protocols, paving the way for advancements in hand surgery and implant development. This biomechanical investigation adds valuable knowledge to the existing literature regarding the evaluation of AdhFix in phalanx fractures of the hand. Future research should focus on repetitive dynamic loading over 300 cycles at 0.2 Hz, simulating one ergotherapy session of 25 min. This could be extended to loading over several ergotherapy sessions. Further research should also be conducted to validate gliding ability and irritation to the surrounding soft tissue. Ongoing research seeks to develop a bioabsorbable construct that removes the need for implant removal, potentially transforming surgical care in pediatric and geriatric patients.

This biomechanical investigation, combined with a subsequent finite element analysis, imparts a profound understanding of internal loads acting in fixations of metacarpal bone fractures during non‐weight‐bearing postoperative rehabilitation exercises. The novel osteosynthesis method AdhFix was found to be able to withstand these exercises, though further evaluation is necessary before AdhFix can be applied in clinical practice.

## Author Contributions

Peter Schwarzenberg, Gordian Banzer, and Tatjana Pastor designed and performed the experiment, analyzed and interpreted data, and drafted the manuscript. Guillaume Patt‐Lafitte and Jérôme Schlatter acquired data and drafted the manuscript. Daniel J. Hutchinson and Michael Malkoch interpreted data and drafted the manuscript. Peter Varga designed the experiment, interpreted data, and drafted the manuscript. All authors have read and approved the final submitted manuscript.

## Ethics Statement

All procedures performed in this study were in accordance with international ethical standards. The specimens were obtained from Science Care (Pheonix, AZ, USA), following approval of the donors for use of their bodies in medical science during their lifetime.

## Conflicts of Interest

MM is involved in Biomedical Bonding AB, which aims to aid patients with adhesive fixators as an alternative to current commercial metal implants. All other authors declare no conflicts of interest.

## Data Availability

The datasets presented in this study can be found in online repositories. The DOI of the repository is: 10.5281/zenodo.15655737.
